# A Practical Application of Genomic Predictions for Mastitis Resistance in Italian Holstein Heifers

**DOI:** 10.3390/ani12182370

**Published:** 2022-09-11

**Authors:** Riccardo Moretti, Stefania Chessa, Stefano Sartore, Dominga Soglia, Daniele Giaccone, Francesca Tiziana Cannizzo, Paola Sacchi

**Affiliations:** 1 Department of Veterinary Science, University of Turin, 10095, Grugliasco, Turin, Italy; 2 Regional Breeder Association of Piedmont (ARAP), 12100 Cuneo, Italy

**Keywords:** Holstein Friesian cattle, genomic selection, heifer selection, genomic index

## Abstract

**Simple Summary:**

Selecting the best animals for farms has always been a fundamental aspect of animal breeding. To assist in the traditional selection performed on phenotypes and to further improve genetic selection as performed in recent years, genomic tools could be applied to select animals early in their productive careers. The aim of this study was to evaluate the application of a commercial genomic tool on animals selected from farms grouped by their average somatic cell count. The obtained results showed that farms with good animal management practices also rear animals with better genomic indexes, probably due to selection on other well-established indexes (e.g., productive traits). Selecting heifers based on their wellness genomic indexes would further improve both their economic value and their disease resistance and resilience.

**Abstract:**

Heifers are a fundamental resource on farms, and their importance is reflected in both farm management and economy. Therefore, the selection of heifers to be reared on a farm should be carefully performed to select only the best animals. Genomic selection is available nowadays to evaluate animals in a fast and economic way. However, it is mainly used on the sire line and on performance traits. Ten farms were selected based on their 5-year records of average somatic cell count and evenly classified into high (>300,000 cells/mL) and low somatic cell count (<150,000 cells/mL). Genomic indexes (regarding both wellness and productive traits) were evaluated in 157 Italian Holstein heifers reared in the selected ten farms (90 from high-cells farms and 67 from low-cells ones). Linear mixed models were fitted to analyze the effects of the abovementioned genomic indexes on related phenotypes. Results have shown that farms classified into low somatic cell count had an overall better animal genomic pool compared to high somatic cell count ones. Additionally, the results shown in this study highlighted a difference in wellness genomic indexes in animals from farms with either a high or a low average somatic cell count. Applying genomic tools directly to heifer selection could improve economic aspects related to herd turnover.

## 1. Introduction

Heifer selection is a critical point in farm management and economy. Since dairy producers traditionally raise most of the heifer calves born inside their farms [1], raising replacement heifers is one of the major costs on dairy farms, secondary only to feed and forage costs for cows [2]. Furthermore, herd turnover (defined as the number of cows culled over 12 months divided by the average population of cows on a farm for the same time [3]) has been observed in the last decade to range from 35 to 50%, compared with historical levels of 25 to 30% [1]. Therefore, being one of the primary factors influencing herd turnover, heifer selection should be carefully performed and guided to keep only the best animals available. Even though traditional selection approaches have played a leading role in animal selection so far, they require too much time to fulfill the contemporary necessities of animal breeding [4]. Advances in molecular biotechnology provided new methodologies and techniques to improve traditional breeding systems [5]. Starting from the study of Meuwissen et al., 2001 [6], microarray technology provided the possibility to simultaneously evaluate multiple markers (e.g., single nucleotide polymorphisms, SNPs) spanning the entire genome, leading to a better prediction of the future performances and breeding values of an animal [6]. Genomic predictions for different traits using microarray technology have been validated and could therefore be used in animal selection, taking into account the known issues when selecting for difficult traits (e.g., low-heritability traits) [7].

From a modern farming perspective, the best animals are not only the best performers but also animals that can proficiently cope with diseases and unideal environments. One way to address health issues is to select animals with an innate genetic resistance to particular diseases [4]. Mastitis is one of the costliest diseases affecting dairy cattle worldwide [8], an infectious disorder responsible for an inflammatory reaction in the mammary gland of cattle [9]. The causes of this infection are typically microorganisms penetrating the mammary gland via the teat canal [10], either transmitted between animals (e.g., *Staphylococcus aureus*) or picked up from the environment (e.g., *Escherichia coli*) [11]. Various studies since the 1950s proved the possible inheritance of the genetic resistance to mastitis and, therefore, the possibility of selecting more resilient animals [12,13]. Mastitis detection relies firstly on clinical diagnosis and secondly on bacteriological analyses and polymerase chain reaction (PCR) assays, which are, however, expensive, time-consuming, and difficult to apply at the population level [14]. Since direct selection for mastitis resistance proved to be poorly efficient because of its low heritability, indirect measures were considered [15]. Therefore, a useful proxy to assess mastitis is milk somatic cell count (SCC) and its log transformation somatic cell score (SCS): an index that evaluates the inflammatory status of the mammary gland, which has been widely used to monitor milk quality and udder health [16], and to select cows with a reduced susceptibility to mastitis [17]. Furthermore, heritability of SCS was assessed to be low-to-moderate (e.g., 0.05 to 0.10) [17,18], thus suggesting the possibility of performing genetic selection [19]. More specifically, genomic selection could be of use when dealing with low-to-moderate heritability traits [20].

The aim of this study was to investigate the possibility of using genomic indexes as a tool for heifer selection regarding their resistance to mastitis. Specifically, in this study, the relationship between genomic indexes and relative phenotypes was analyzed during the first lactation of Italian Holstein cows raised on ten different farms.

## 2. Materials and Methods

### 2.1. Farms

A total of 10 Italian Holstein farms were selected in the provinces of Turin and Cuneo (Piedmont region, Northern Italy). The selection criterion was based on the average SCC calculated for the previous 5-year interval (2014–2019). Farms with an average 5-year SCC lower than 150,000 cells/mL were classified as low SCC (LS), while farms with an average 5-year SCC higher than 300,000 cells/mL were classified as high SCC (HS). The farms were evenly selected among the two groups (i.e., 5 LS and 5 HS). The two classes (i.e., LS and HS) were built on the two divergent tails of the farm’s SCC distribution to amplify the differences between them and to highlight the results obtained with the statistical modeling approach, similarly to what was achieved by Moretti et al., 2021 [21].

In the selected farms, genomic selection was performed on the sires only and mainly taking into account performance genomic indexes (e.g., milk yield and composition traits). No selection was made on females or on wellness traits.

### 2.2. Animals

A total of 157 Italian Holstein heifers were selected on the 10 farms: 67 heifers from LS farms and 90 from HS ones. On each farm, around 15% of the available heifers were selected. Ear tissue for the genomic analysis was sampled in conjunction with the application of ear tags; therefore, no ethical approval was required for the sampling of biological material. The animals were subsequently followed during their first lactation (considered between 5 and 305 days in milk), and data on milk yield, milk composition (i.e., fat, protein, and lactose percentage), and SCC were collected during routine milk recording procedures (monthly functional controls) by the staff of the Regional Breeder Association of Piedmont.

### 2.3. Genomic Analysis and Indexes

Genomic testing was performed by the Clarifide Plus genomic testing system (Zoetis Genetics, Kalamazoo, MI, USA). This system offers a comprehensive package of trait predictions, economic, and wellness indexes. Specifically, two wellness indexes and three productive traits indexes were analyzed: the former are SCS and Z_MAST (i.e., Zoetis Mastitis Index); the latter are milk, fat, and protein production indexes. The first wellness index, SCS, is an indirect predictor of mastitis. When used to compare two animals, lower values indicate a lower average SCC value of the indexed animal compared to the other. The second wellness index, Z_MAST, is a specific mastitis index developed by Zoetis. When used to compare two animals, higher values indicate the animal with a lower mastitis risk. The productive indexes (i.e., milk, fat, and protein) give an indication regarding the genetic differences in the relative trait yield during a standard lactation of 305 days.

### 2.4. Statistical Analyses

All the statistical analyses and tests were performed in an R software environment [22]. Summary statistics were calculated by grouping the samples by the farm’s 5-year SCC classification (i.e., LS and HS), and differences between means were tested using the *t*-test. Pearson’s correlation was also calculated. The following linear mixed model was fitted using the *lmerTest* R Package [23] to evaluate the effects of different variables on the phenotypes of interest (i.e., milk yield and SCC):

$$Pheno_{ijk}=µ+Geno_{i}+DIM_{i}+Farm_{group}{}_{j}+Age_{i}+Animal_{i}+\;Test\_day_{k}+HYS_{i}+\varepsilon_{ijk}$$

where *Pheno_ijk_* is the phenotypic value (i.e., SCC and milk yield) for each animal; G*eno_i_* is the fixed effect of the related genomic index of the *i*-th animal (continuous variable); *DIM_i_* is the fixed effect of Days in Milk (DIM) for the *i*-th animal (categorical variable: I: DIM < 45 days; II: 45 ≤ DIM < 90 days; III: DIM ≥ 90 day—the thresholds were selected to identify the three phases before, around, and after the lactation peak); *Farm_group_j_* is the fixed effect of the *j*-th farm classification based on the 5-year SCC average (categorical variable: LS and HS); *Age_i_* is the fixed effect of the age at first parturition of the *i*-th animal (continuous variable); *Animal_i_* is the random effect of the *i*-th animal (in order to take into account the correlation between measurements recorded from the same animal); *Test_day_k_* is the random effect of the *k*-th sampling day; *HYS_i_* is the random effect of the Herd-Year-Season contemporary group for the *i*-th animal (categorical variable, 39 levels, year referred to date of first calving event ranging from 2019 to 2021); *ε_ijk_* the random residual effect. Fixed effects descriptive statistics (i.e., mean, SD, range, number of observations) are reported in Table S1 (Supplementary Materials). Conditional R^2^ for the mixed model was calculated using the *r.squaredGLMM* function from the *MuMIn* R package [24], which returns revised statistics based on Nakagawa et al., 2017 [25]. Different sub-models were tested by individually removing non-significant predictors from the two starting models, and the best model for each of the two phenotypes was selected by comparing the conditional R^2^, the correlation between observed and predicted values, and the Root Mean Square Error (RMSE).

## 3. Results

### 3.1. Descriptive Statistics

#### 3.1.1. Genomic Indexes

Genomic indexes were calculated by Zoetis Clarifide Plus (Zoetis Genetics, Kalamazoo, MI, USA) genomic testing system. Each animal index was grouped by farm classification and then averaged. Differences in the means of the five genomic indexes (i.e., SCS, Z_MAST, Milk, Fat, Protein) for the two groups were tested with a *t*-test, and the results are reported in Table 1.

Regarding the wellness indexes (i.e., SCS and Z_MAST), the difference between the mean SCS index in the two groups was statistically significant (*t*-test, *t* = −2.863, df = 121.42, *p*-value 0.005), while no statistically significant differences were observed between the mean Z_MAST index in the two groups. Regarding the productive traits’ indexes, the difference between the mean Fat index in the two groups was statistically significant (*t*-test, *t* = 5.378, df = 114.09, *p*-value < 0.001), as well as the one between the mean Protein index (*t*-test, *t* = 4.097, df = 129.18, *p*-value < 0.001), while no statistically significant differences were observed between the mean Milk index in the two groups.

Pearson’s correlation was calculated between the wellness genomic indexes and their related phenotypes. Specifically, the correlation between the SCS index and the SCC phenotype was 0.22 (95% CI = 0.17 to 0.27, *p*-value < 0.001), while the correlation between the Z_MAST index and the SCC phenotype was −0.10 (95% CI = −0.16 to 0.05, *p*-value < 0.001). Similarly, a correlation was calculated between productive traits genomic indexes and their related phenotype. The correlation between the milk index and milk yield phenotype was 0.27 (95% CI = 0.22 to 0.32, *p*-value < 0.001); the correlation between the protein index and protein % phenotype was −0.05 (95% CI = −0.10 to 0.004, *p*-value = 0.070); the correlation between the fat index and fat % phenotype was 0.18 (95% CI = 0.13 to 0.23, *p*-value < 0.001). Lastly, correlations between indexes were calculated: SCS and Z_MAST −0.59 (95% CI = −0.63 to −0.56, *p*-value < 0.001); SCS and Milk 0.009 (95% CI = −0.04 to 0.06, *p*-value = 0.74); SCS and Protein −0.14 (95% CI = −0.19 to −0.09, *p*-value < 0.001); SCS and Fat −0.17 (95% CI = −0.22 to −0.12, *p*-value < 0.001); Z_MAST and Milk −0.05 (95% CI = −0.11 to 0.002, *p*-value = 0.057); Z_MAST and Protein −0.09 (95% CI = −0.15 to −0.04, *p*-value < 0.001); Z_MAST and Fat −0.05 (95% CI = −0.10 to 0.006, *p*-value = 0.084); Milk and Protein 0.54 (95% CI = 0.50 to 0.57, *p*-value < 0.001); Milk and Fat 0.25 (95% CI = 0.20 to 0.30, *p*-value < 0.001); Protein and Fat 0.73 (95% CI = 0.71 to 0.76, *p*-value < 0.001).

#### 3.1.2. Productive Traits

Milk data from functional controls of the selected cows at their first lactation recorded from January 2020 to December 2021 were grouped by SCC classification (i.e., LS and HS) and subsequently analyzed. A data quality check was performed, and observations from animals with missing birth and first calving dates were removed, along with observations from animals with less than three functional controls. After data pruning, a total of 143 animals (60 and 83, LS and HS, respectively) were subjected to further analyses. A total of 1400 observations were therefore available in the final dataset (536 and 862, LS and HS, respectively), with an average number (mean ± SD) of observation per animal of 9.79 ± 2.35 (range = 3 to 18) and an average number (mean ± SD) of observations per farm of 140.00 ± 59.11 (range = 52 to 258). The average number of animals per farm was 14.30 ± 5.56 (range = 7 to 25). The animals’ age at functional control ranged from 1.80 to 4.21 years (2.69 ± 0.41 years, mean ± SD).

Differences in the milk yield and composition for the means of the two groups were tested with a *t*-test, and the results are reported in Table 2.

The difference between the SCC means in the two groups was statistically significant (*t*-test, *t* = 5.589, df = 1035.4, *p*-value < 0.001), as well as the one between the mean milk yield (*t*-test, *t* = −6.403, df = 945.69, *p*-value < 0.001), mean fat % (*t*-test, *t* = −8.815, df = 1106.4, *p*-value < 0.001), and mean lactose % (*t*-test, *t* = 3.635, df = 1168.6, *p*-value = 0.0003).

### 3.2. Mixed models Analysis

The pruned dataset consisting of 118 subjects was randomly split into a training and a testing subset, with a 4:1 ratio (i.e., 80% of the animals used for training and 20% for testing). To obtain a better statistical result, random splitting into a training and testing dataset was performed ten times, and the total results were averaged in a bootstrapping-like procedure. The previously described model was therefore trained using only the data in the training subset. Non-significant variables were singularly removed, and each reduced model was compared to others through *anova* function in R. The results of the subsequent validation phase are described in the following paragraphs.

#### 3.2.1. SCC

Following the ten iterations of the mixed model applied to the model, excluding the age of the animal at first parity, the best model was selected, and the results are reported in Table 3. The random animal effects variances and standard deviations are reported in section (A) of Table 3, while the fixed effects estimates, standard error of the means, and significance are reported in section (B).

The intercept of the model (i.e., the value of SCC phenotype with genomic index = 0, DIM class = I, and 5-years SCC average = LS) was not statistically significant and, therefore, not different from 0 (*p*-value 0.583). The effect of genomic index was statistically significant (*p*-value = 0.001), and the DIM class II was statistically different from class I (*p*-value 0.002), while the DIM class III was not statistically different from class I (*p*-value 0.965), and the HS class was not statistically different from LS class (*p*-value 0.061), although close to the 0.05 threshold. Conditional R^2^ for the selected mixed model was 39.57%. The selected model was therefore fitted to the testing dataset to check its predicting performances. The correlation between observed and predicted data was 0.19 (*p*-value = 0.001), and the RMSE was 1.39.

#### 3.2.2. Milk Yield

Following the ten iterations of the mixed model applied on milk yield data, the model excluding the age of the animal at its first parity was selected as the best model, with an average conditional R^2^ of 83.13%, an average correlation between observed and predicted data of 0.70, and an average RMSE of 55.86. The specific results of the best iteration are reported in Table 4. The random animal effects variances and standard deviations are reported in section (A) of Table 4, while fixed effects estimates, standard error of the means, and significance are reported in section (B).

The intercept of the model (i.e., the value of milk yield phenotype with genomic index = 0, DIM class = I, 5-years SCC average = LS, and age of the animal at first parity = 0) was statistically significant and therefore different from 0 (*p*-value < 0.001). Both the effects of the genomic index and DIM classes were statistically significant (*p*-values < 0.001), while the effects of farms grouped by 5-year SCC average and age of the animal at first parity were not statistically significant. Conditional R^2^ for the selected mixed model was 80.33%. The selected model was therefore fitted to the testing dataset to check its predicting performances. The correlation between observed and predicted data was 0.71 (*p*-value < 0.001), and the RMSE was 61.98.

## 4. Discussion

The definition of an SCC threshold to classify milk status (i.e., intramammary infection, clinical and subclinical mastitis) is a controversial subject [26]. Starting in the 1960s, different thresholds were proposed based on the current knowledge of the topic in those years. In recent years, an SCC < 100,000 cells/mL (in addition to the absence of microorganisms isolated from the sample) was suggested to define healthy quarters [16], while an SCC ≥ 200,000 cells/mL (without clinical changes to milk composition and properties) was suggested to define subclinical mastitis [27], even if a tolerance range up to 400,000 cells/mL was recommended for practical reasons [16]. Regarding composite milk samples (i.e., four-quarters of an animal), a threshold of 150,000 cells/mL was suggested [28]. According to these suggestions from the literature, the two thresholds used in this study (i.e., SCC < 150,000 cells/mL and SCC > 300,000 cells/mL) were selected to enhance the differences between the two groups. As shown in the results of this study, the average SCC for cows at their first lactation is generally lower than the selected thresholds (i.e., SCC > 300,000 cells/mL and SCC < 150,000 cells/mL for HS and LS classes, respectively). Nevertheless, the difference between the two groups (189.82 and 81.52 kcells/mL, for HS and LS classes, respectively) remained significantly different (*p*-value < 0.001) and justified the dichotomous classification of the farms and the relative animals.

The wellness indexes were analyzed and showed the following results: the SCS index had a statistically significant difference (*p*-value = 0.005) between the means of LS and HS farms, while the Z_MAST index did not show statistically significant differences in the two groups (*p*-value = 0.857). This could be due to the fact that the Italian national breeders’ association for the Italian Holstein breed added an SCS index to the national selection scheme in the first years of the XXI century, while a general udder health index was added only recently. Therefore, even if the farmers did not actively select animals with better SCS indexes, the Italian population changed accordingly to the national selection scheme. The Pearson’s correlation found between the SCS and Z_MAST index is consistent with studies found in the scientific literature that support the use of SCC as an indicator for mastitis [15] (the two indexes resulted inversely correlated because their measurement scale is oriented in the two opposite directions). The phenotypic differences in SCC observed on LS and HS farms were confirmed on a genomic indexes level, showing that breeders of LS farms are effectively improving their herds and not only obtaining this low SCC level due to good farm and animal management. All the functional control parameter means between LS and HS farms (excluding protein %) were statistically different. Specifically, mean milk yield and fat % were higher in animals from LS farms than in animals from HS farms, while mean lactose % and SCC were lower in animals from LS farms than in animals from HS farms. Our results are consistent with both scientific and empirical evidence that indicate an inverse relationship between milk yield and SCC [29]. Regarding genomic indexes, statistically significant differences between them were observed. In the productive traits’ indexes, only the Milk one did not differ between animals from LS and HS farms (*p*-value = 0.742). Even though farmers’ preferences are known to be heterogeneous [30], the Milk index is one of the main indexes considered by them when selecting their animals since it is the one having a major short-term economic impact. Given this general interest for the majority of dairy cattle breeders, the absence of a statistical difference between the Milk index on LS and HS farms was expected (meaning that, despite the differences in animal and farm management, breeders in both groups selected their animals mainly based on this trait).

The fitted mixed model showed that there is a large variability among the animals for both milk yield (23.72% of the total explained variance of the model) and SCC traits (27.96% of the total explained variance of the model). The milk yield model performed better than SCC one, both when fitting observed data (Conditional R^2^ for milk yield and SCC models were 80.33 and 39.57%, respectively) and when predicting data using the testing dataset (observed-predicted data correlation for milk yield and SCC models were 0.71 and 0.19, respectively). Predictors included in our study came mainly from functional controls and, therefore, better describe variations in milk yield. Regarding the milk yield predicting model, although not statistically significant, both 5-year average SCC classification and age at first parity were not excluded from the model. Removing these two predictors reduced both the conditional R^2^ and the correlation between observed and predicted values and increased the RMSE of the models. Differently, regarding the SCC predicting model, while both 5-year average SCC classification and age at first parity were not statistically significant, only the latter was excluded from the model. Indeed, removing the age predictor reduced the conditional R^2^ from 39.63 to 39.57%; however, both the observed/predicted correlation and RMSE slightly improved (respectively, from 0.17 to 0.19, and from 1.40 to 1.39). Furthermore, the *anova* function in R showed a statistically significant difference (*p*-value = 0.049) between the models with and without 5-year average SCC classification, indicating the former as the best one.

## 5. Conclusions

Welfare of heifers and their selection for improved resistance to mastitis was the focus of this study. Genomic indexes were evaluated on a sample of Italian Holstein heifers and subsequently related to their relative phenotypes. The differences in the abovementioned indexes evaluated in animals from farms with different levels of average SCC showed that it is possible to use them to classify animals and to potentially perform selection. Furthermore, the results from this study showed that a bland selection has already happened even if no direct selection had been performed on females since animals on LS farms had, on average, better genomic indexes than animals in HS farms. In addition to the good farming and management practices, a further genomic selection, based on wellness indexes other than the classical productive ones, could greatly benefit to cattle breeders. Furthermore, while in traditional farms, the genetic improvement is pursued, acting mainly on the sire-daughter line, applying genomic tools directly to heifer selection is feasible and could improve economic aspects related to herd turnover.

## Figures and Tables

**Table 1:** Mean values of genomic indexes from Zoetis Clarifide Plus genomic testing system. Differences were statistically tested through *t*-test (SEM and *p*-value are reported, where available).

Variables	LS ^1^ (SEM) *n* = 60	HS ^1^ (SEM) *n* = 83	*p*-Value
SCS	2.91 (0.02)	2.98 (0.02)	0.005
Z_MAST	99.71 (0.66)	99.86 (0.49)	0.857
Milk	49.07 (61.92)	23.07 (48.11)	0.742
Fat	21.93 (3.58)	−2.16 (2.65)	<0.001
Protein	13.54 (2.35)	0.54 (2.11)	<0.001

^1^ SCC = Somatic Cell Count; LS = Low Somatic Cell Count (<150,000 cells/mL); HS = High Somatic Cell Count (>300,000 cells/mL). The number indicated with “*n* = ” below the LS and HS classes is the number of animals in the relative group.

**Table 2:** Mean values of milk data from functional controls grouped by SCC ^1^ classification (i.e., LS ^1^ and HS ^1^). Differences were statistically tested through *t*-test (SEM and *p*-value are reported, where available).

Variables	LS ^1^ (SEM) *n* = 60	HS ^1^ (SEM) *n* = 83	*p*-Value
# Observations	538	862	-
Milk yield (kg)	32.07 (0.37)	29.25 (0.23)	<0.001
Fat %	4.27 (0.04)	3.80 (0.03)	<0.001
Protein %	3.49 (0.02)	3.52 (0.01)	0.203
Lactose %	4.90 (0.01)	4.93 (0.01)	<0.001
SCC^1^	81.52 (6.36)	189.82 (18.11)	<0.001

^1^ SCC = Somatic Cell Count (cells * 1000/mL); LS = Low Somatic Cell Count (<150,000 cells/mL); HS = High Somatic Cell Count (>300,000 cells/mL). The number indicated with “*n* = ” below the LS and HS classes is the number of animals in the relative group.

**Table 3:** (**A**) Random effects, variances, and standard deviations (SD). (**B**) Fixed effects estimates, standard error of the means (SEM) and significance by *p*-value (SCC).

**(A)**			
**Random Effect**	**Variance**	**SD**	**Explained Variance %**
Animal_i_	0.722	0.850	27.96
Test_day_k_	0.063	0.251	2.45
HYS_i_	0.097	0.312	3.77
Residual	1.700	1.304	65.82
**(B)**			
**Fixed effect**	**Estimate**	**SEM**	***p*-value**
Intercept	−1.093	1.986	0.583
Geno_i_ (SCS)	2.312	0.682	0.001
DIM: II	−0.542	0.176	0.002
DIM: III	−0.006	0.135	0.965
Farm_group_j_: HS	0.445	0.229	0.061

**Table 4:** (**A**) Random effects, variances, and standard deviations (SD). (**B**) Fixed effects estimates, standard error of the means (SEM) and significance by *p*-value (Milk yield).

**(A)**			
**Random Effect**	**Variance**	**SD**	**Explained Variance %**
Animal_i_	1480	38.47	23.72
Test_day_k_	1790	42.31	28.69
HYS_i_	1657	40.71	26.56
Residual	1312	36.22	21.03
**(B)**			
**Fixed effect**	**Estimate**	**SEM**	***p*-value**
Intercept	248.67	45.49	<0.001
Geno_i_ (milk yield)	0.37	0.09	<0.001
DIM_i_: II	25.95	5.31	<0.001
DIM_i_: III	22.02	4.75	<0.001
Farm_group_j_: HS	−20.05	16.93	0.244
Age_i_	16.50	19.52	0.400

## Data Availability

The data are contained within the article and are available on request from the corresponding author.

## Supplementary Materials

The following supporting information can be downloaded at: https://www.mdpi.com/article/10.3390/ani12182370/s1, Table S1: Descriptive statistics of all fixed effects included in the model in the context of 10 farms (mean, SD, range, number of observations).
