# Supplementary material for: A Practical Application of Genomic Predictions for Mastitis Resistance in Italian Holstein Heifers
Source: Animals (Basel). 2022 Sep 11;12(18):2370. doi: 10.3390/ani12182370 (PMC9494965; doi:10.3390/ani12182370)
Supplement: Supplementary file 1 [file animals-12-02370-s001.zip › animals-1837394-supplementary.pdf]

SUPPLEMENTARY MATERIALS

TABLE S1. Descriptive statistics of all fixed effects included in the model in the context of 10 farms (mean, SD, range, number of observations).

Model	Fixed effect[1]	Number of classes[2]	Mean	SD	Range	Number of observations
SCC	Geno (SCS)	-	2.95	0.14	2.55 – 3.33	1118
	DIM	3	192.1	122.1	5 – 305	
	Farm group	2	-	-	-	
	Age	-	2.15	0.26	1.65 – 3.31	
Milk Yield	Geno (Milk yield)	-	4.52	44.45	-92.2 – 99.1	1111
	DIM	3	196.0	124.9	5 – 305	
	Farm group	2	-	-	-	
	Age	-	2.15	0.26	1.65 – 3.31	

[1] *Geno* is the fixed effect of the related (i.e., SCS or Milk Yield) genomic index; *DIM* is the fixed effect of Days in Milk; *Farm group* is the fixed effect of the farm classification based on the 5-years SCC average; *Age* is the fixed effect of the age at first parturition. Being *Farm group* a categorical variable, only number of observations is shown.

[2] Number of classes is specified for categorical fixed effects only.
